# Assessment of Oral Health-Related Quality of Life in Children with Leukemia and Gingival Inflammation

**DOI:** 10.3390/jpm16020084

**Published:** 2026-02-02

**Authors:** Alina Adumitroaie, Vasilica Toma, Minerva Codruta Badescu, Daniel Cioloca, Aurelia Spinei, Nura Jdid, Mioara Florentina Trandafirescu, Carmen Ecaterina Leferman, Liliana Georgeta Foia

**Affiliations:** 1 Grigore T. Popa University of Medicine and Pharmacy Iasi, 700115 Iasi, Romania; alina.adumitroaie@umfiasi.ro (A.A.); minerva.badescu@umfiasi.ro (M.C.B.); daniel-petru.cioloca@umfiasi.ro (D.C.); georgeta.foia@umfiasi.ro (L.G.F.); 2 Nicolae Testemitanu State University of Medicine and Pharmacy, MD-2004 Chisinau, Moldova

**Keywords:** oral health-related quality of life, children, leukemia, gingivo-periodontal alterations

## Abstract

**Background/Objectives**: Oral health-related quality of life (OHRQoL) is a complex topic, encompassing the medical, functional and psychosocial aspects of well-being, especially in the context of systemic conditions that can trigger oral cavity impairment. While this subject has been extensively investigated in adults, evidence remains limited in pediatric populations, particularly in children with leukemia who are at high risk for oral complications related to the disease itself and its treatment. Moreover, children and parent perceptions of oral health are essential for guiding preventive and personalized therapeutic strategies, yet they are poorly explored in this clinical context. The objective of this study was to assess OHRQoL in children with leukemia and gingival inflammation, and compare it with that of children without this systemic condition. **Methods**: This observational, cross-sectional, case–control study was conducted on 99 subjects, divided into two groups: the study group *n* = 49 leukemia subjects and the control group *n* = 50 subjects without oncologic pathology. Clinical examination of all subjects was performed and oral health status was evaluated using Oral Health Index-Simplified (OHI-S) and Gingival Index (GI). Parents filled out a personalized exploratory questionnaire, adapted after established scales, designed to capture the child’s perceived impact of certain leukemia-related gingivo-periodontal alterations, including pain, ulcerations, gingival bleeding and xerostomia. Data were analyzed using descriptive statistics, Pearson’s Chi-square test and comparative graphical analyses (IBM SPSS Statistics 26). **Results**: Children with leukemia reported higher frequencies of xerostomia, ulcerations and gingival bleeding compared to children in the control group, with xerostomia showing a suggestive association to gingival inflammation. Oral hygiene status of children in the leukemia group was generally better among children receiving parental assistance during brushing or those practicing dental flossing. Comparative graphical analyses showed differences in symptom reporting and oral hygiene support between groups. **Conclusions**: The results suggest that xerostomia seemed to align with gingival inflammation in children with leukemia, while parental assistance and dental flossing seemed to be associated with better oral hygiene status. Our findings also support the need for developing standardized, disease-oriented scales of evaluating OHRQoL, as well as individualized oral care and continuous monitoring in order to improve oral health-related quality of life in this vulnerable pediatric population.

## 1. Introduction

The multidimensional concept of oral health-related quality of life (OHRQoL) should become a routine instrument for the evaluation of a patient’s well-being, to be examined in the dental office through a holistic point of view. The biological, socio-cultural and psychological model on which this construct relies includes the analysis of the oral health status from a clinical, functional and psychological standpoint, but also the general health-related quality of life assessment, by recognizing the environmental or contextual factors’ involvement [1].

When evaluating oral health-related quality of life, the focus is to be put on subjective experiences and states of the patient, making a shift from medical standards of evaluating oro-dental health status to other criteria, similar to the bio-psycho-social health pattern [2]. Although this concept has been extensively studied in adults, literature is relatively scarce concerning the evaluation of oral health-related quality of life in children, particularly in children with leukemia.

In pediatric populations, OHRQoL assessment poses certain challenges that mostly arise from children’s cognitive and psycho-behavioral development differences. Despite this, the evaluation is a crucial tool as it offers individualized perspectives from children and parents, supporting the finding and prioritization of dental care needs, especially in vulnerable groups [3,4,5].

Oral health-related quality of life in children and adolescents can be assessed through certain scales, among which the most frequently used in the literature are as follows: Early Childhood Oral Health Impact Scale (ECOHIS), Child—Oral Impact on Daily Performance (Child-OIDP), Child Oral Health Impact Profile (COHIP), Paediatric Oral Health-related Quality of life Questionnaire (POQL) [6,7,8,9]. ECOHIS is commonly used in children and includes parent-reported outcomes, focusing on the impact of oral conditions on both the child and family, particularly in relation to dental caries [10,11,12,13]. Child-OIDP seems more suitable for self-reporting by older children and adolescents, evaluating the functional, emotional and social impact of oral and dental disorders [14,15]. COHIP offers a broader perspective by addressing oral and maxillofacial disturbances alongside psychosocial aspects [16], while POQL allows a comprehensive evaluation of oral health status in general and vulnerable pediatric populations [17,18].

Children with leukemia often present gingival and periodontal alterations that can significantly affect their daily functioning and psychosocial well-being, such as increased gingival inflammation, bleeding and ulcerations [19,20]. However, current oral health-related quality of life tools do not include a standardized pediatric instrument specifically addressing the impact of periodontal health on the quality of life, highlighting the need to develop a dedicated scale to better capture the functional and psychosocial burden of periodontal alteration in vulnerable groups such as children suffering from leukemia.

The literature highlights several efforts to develop periodontal health-related quality of life instruments in adults and children, including adaptations of the OHIP questionnaire [21,22], children self-evaluation tools focused on their gingival health and oral hygiene behaviors [23], and also comprehensive tools such as the “Gum Health Experience Questionnaire” [24], all of which aim to capture the functional and psychosocial impacts of periodontal conditions on the quality of life.

Despite these developments, a clear gap remains, as no validated instrument currently addresses the specific impact of gingivo-periodontal alterations on the quality of life in children with leukemia. The clinical and psychosocial consequences of these changes remain insufficiently understood, which underlines the need for targeted research in this vulnerable population.

Although oral health-related quality of life in children has been widely studied and certain evaluation tools to assess the impact of gingival changes exist, little research has specifically examined gingivo-periodontal aspects in pediatric leukemia patients, where such periodontal alterations might present with unique consequences. This study aims to evaluate OHRQoL in children with leukemia from a gingivo-periodontal perspective.

## 2. Materials and Methods

**Figure 1:**
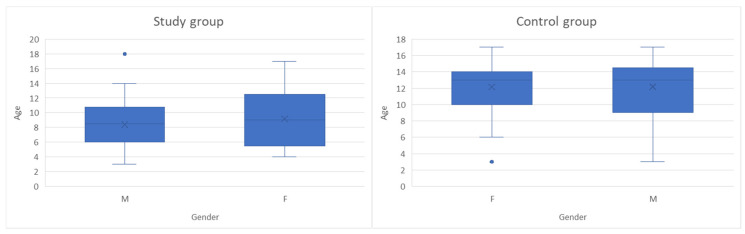
Age and gender distribution of children in both groups (The box plots represent the median (horizontal line), interquartile range (box), and minimum–maximum values excluding outliers (whiskers). The “×” symbol indicates the mean age. Blue dots represent individual outlier values).

This study was an observational, cross-sectional, case–control study conducted on 99 children with ages between 3 and 18 years old, monitored in “St. Mary” Clinical Emergency Hospital for Children in Iasi between October 2024 and May 2025, divided into two groups: 49 subjects were included in the study group and 50 children in the control group. Figure 1 presents the age and gender distribution for children in both groups. According to their general health state and gender, the study group comprised 28 boys and 21 girls diagnosed with leukemia, while the control group consisted of 21 boys and 29 girls without oncological pathology, hospitalized for other non-inflammatory conditions (most of which were non-surgical abdominal pain syndromes and accidental drug intoxications). The inclusion and exclusion criteria for both subject groups are presented in Table 1; sample size calculation was not performed due to the limited number of children with oncologic pathology. The small and specific study sample made formal power calculations and gender-based stratification impractical, and the study was designed as an exploratory observational analysis rather than a hypothesis-driven trial. No stratification or adjustment was made for potential confounding effects related to disease subtype, treatment phase or time since diagnosis, as children in the study group included a heterogenous distribution of leukemia subtypes (acute lymphoblastic, acute myeloid, chronic myeloid) and cancer treatment phases (induction, consolidation, maintenance).

Clinical examination and evaluation of the oral hygiene and gingival inflammation status were performed using the Oral Hygiene Index (OHI) and Gingival Index (GI). Despite formal reproducibility, statistics were not computed, examiner reliability was addressed through calibration performed under the supervision of an experienced periodontist, with repeated assessments performed until scoring consistency was established.

Parents completed a personalized exploratory questionnaire, designed to evaluate oral health-related quality of life in children. This instrument was adapted from established questionnaires (ECOHIS, Child-OIDP, COHIP and POQL) by selecting relevant aspects and adding targeted items addressing gingivo-periodontal alterations commonly observed in pediatric leukemia. The main focus was put on periodontal changes associated to the oncological pathology, by evaluating child’s perception regarding pain, ulcerations, gingival bleeding or xerostomia. The questionnaire also evaluated psychosocial components by assessing the perceived impact of oral health on the child’s daily activities alongside behavioral items related to oral hygiene practices, such as toothbrushing frequency and the use of adjunctive hygiene methods (mouth rinses and dental floss). The final questionnaire comprised two sections: (1) ten closed-ended items recording oral health, answered using Likert-type frequency scale responses (“Very often”, “Often”, “Quite often”, “Rarely”, “Never”) and (2) five open-ended questions addressing oral hygiene behaviors. For children in the study group, one additional open-ended question was included, to collect information on oral hygiene instructions received during the oncological treatment and the source of this information. Items were completed by parents and written informed consent was obtained from all participants prior to answering the questionnaire. Each question was explained to respondents using appropriate language to ensure clarity and high response rates; clarifications were offered wherever uncertainties arose. The questionnaires are presented in Figures S1 and S2 from Supplementary Materials.

Given its exploratory nature and the fact that it was self-developed, the questionnaire was not formally tested or subjected to psychometric validation, and no data on reliability or cultural and linguistic adaptation were obtained. To analyze the distribution of the main study variables, we used comparative summary tables, whereas associations between categories were evaluated using Pearson’s Chi-Square test; correspondence analyses, with correspondence graphics for multivariate relationships exploration and potential patterns among categories identification, were also performed. Given the relatively small sample size, *p*-values < 0.10 were considered indicative of exploratory patterns for certain indicated items, rather than evidence of formal statistical significance. All statistical analyses were conducted using the IBM SPSS Statistics package, version 26.0 (IBM, Armonk, NY, USA).

Carried out in accordance with the Declaration of Helsinki, this study was approved by the Ethical Committee of the “St. Mary” Clinical Emergency Hospital for Children in Iasi and the Ethical Committee of the “Grigore T. Popa” University of Medicine and Pharmacy from Iasi (approval no. 34161/14 October 2024 and approval no. 477/16 September 2024). Informed consent was obtained from the parents of all children participants in this study.

## 3. Results

The comparative analysis, when evaluating responses from the first set of questions (the 10 items related to oral health as perceived by subjects), exhibits significant differences for most variables related to oral symptoms, for a 5% threshold (Table 2). Children from the control group more frequently reported complaints related to their smile, peer difficulties caused by oral health issues, pain in the oral cavity, sleep disturbances, concentration difficulties in school or school absenteeism. Burning sensation was more frequently claimed by children from the control group compared to no reports in children from the study group, indicating a statistically significant difference for a 10% threshold. A meaningful discrepancy has also been stated concerning ulceration reports, as children in the control group predominantly responded with “Never” and “Rarely”, while children in the study group chose responses from the “Quite often”, “Often” and “Very often” categories. Regarding dry mouth, respondents in the control group more frequently reported symptoms, while children in the study group predominantly reported “Never” responses; the difference is significant for a 10% threshold.

When evaluating responses from the second set of questions (the 5 items related to oral hygiene measures), we observed fewer statistically significant differences (Table 3). Regarding dental brushing frequency, after eliminating certain isolated responses (“Never brushes teeth”), there were no differences between the two groups, both having similar brushing habits of 1 or 2 times daily. For parent assistance during brushing, the difference is statistically significant for a 10% threshold: 36.7% of children with leukemia are assisted by parents, compared to only 20% of the children in the control group. In our survey, the type of toothbrush does not seem to be a differentiating factor, as approximately 60% of children in both groups use manual brushes, followed by approximately 17% that handle electric toothbrushes. No statistically significant differences have been noticed in the case of mouthwash use, as well: approximately half of the children never use mouthwash, while the rest use it either occasionally or daily. For dental flossing, the difference is statistically significant for a 10% threshold: 24% of children in the control group use dental floss, compared to only 10.2% of children in the leukemia group.

We evaluated possible associations between gingival inflammation status assessed by GI (Gingival Index after Löe and Silness) and oral health as perceived by subjects in both groups, using the 10 questions of the first section of the questionnaire.

For children in the study group, most differences were not statistically significant (Table 4). The only significant relationship was found for xerostomia; almost two thirds of children with mild inflammation status reported never having experienced dry mouth, children with moderate gingival inflammation shifted the distribution to “Never” and “Rare” responses, while children with severe gingival inflammation claimed more “Rare” and “Quite often” responses.

For children in the control group, the overall results show no statistical significance between questionnaire responses regarding self-perceived oral health and clinically assessed gingival inflammation status. Response distribution is relatively uniform between mild and moderate gingival inflammation status (Table 5).

**Figure 2:**
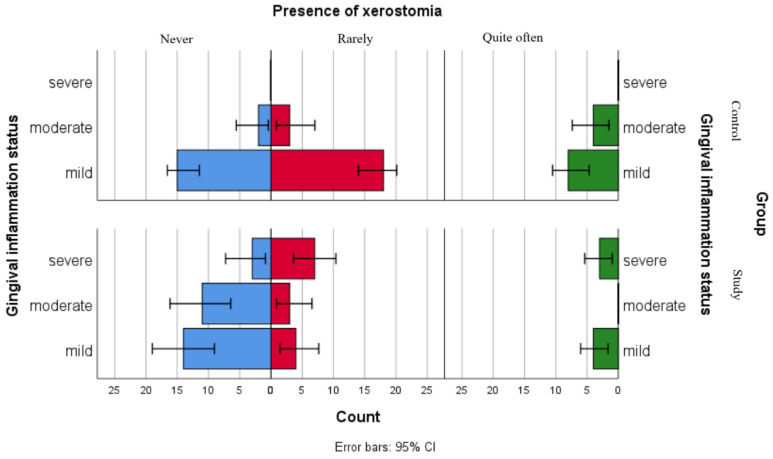
Comparative analysis of xerostomia presence and association with gingival inflammation status categories.

**Figure 3:**
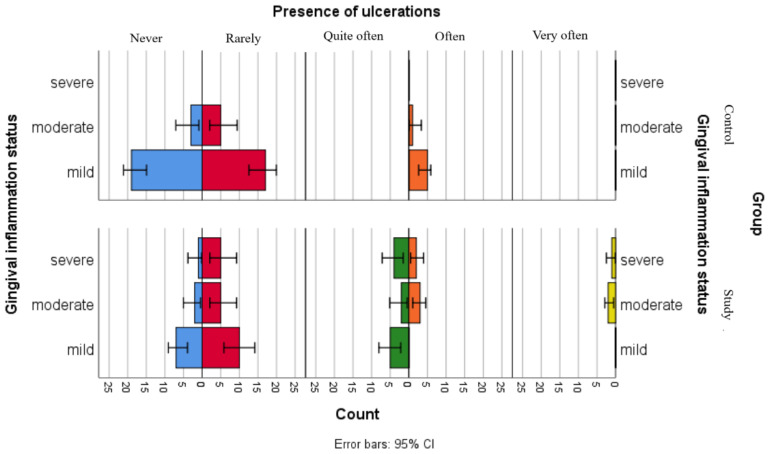
Comparative analysis of ulceration presence and association with gingival inflammation status categories.

**Figure 4:**
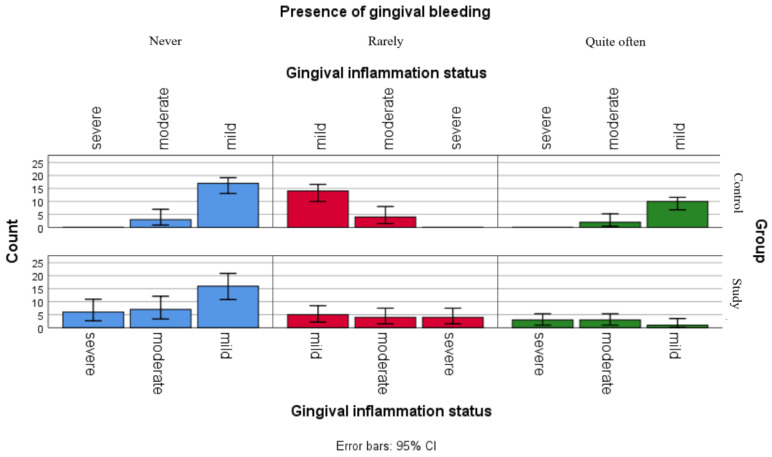
Comparative analysis of gingival bleeding and association with gingival inflammation status categories.

Although the separate analyses of associations between gingival inflammation status and self-reported oral health status showed no statistically significant relationships for children in the control group and only one significant association with xerostomia for children in the study group, the comparative graphical analyses present the distributions of xerostomia, ulcerations and gingival bleeding in both groups (Figure 2, Figure 3 and Figure 4).

Figure 2 presents the distribution of xerostomia reports among gingival inflammation status categories in both groups. The results suggest that children in the study group reported xerostomia more frequently as compared to children in the control group across all gingival inflammation categories, including those within the mild category. For children with leukemia from the moderate and severe gingival inflammation status category, this difference was more pronounced.

Figure 3 illustrates the distribution of the reported ulcerations among gingival inflammation statuses in both groups. The results suggest that children in the control group have less declared the presence of ulcerations, including those in the moderate gingival inflammation category, while children with leukemia seem to more frequently report ulcerations, across all gingival inflammation categories.

Figure 4 presents the distribution of reported gingival bleeding among gingival inflammation statuses categories in both groups. The graphic points that children in the study group seem to be more frequently report gum bleeding, with relatively similar frequencies observed across all inflammation categories, while respondents in the control group presented more variable reporting frequencies.

We evaluated possible associations between oral hygiene status assessed by OHI-S (Oral Health Index-Simplified modified after Greene-Vermilion) and oral hygiene as perceived by subjects in both groups, using the 5 questions of the second section of the questionnaire.

For children in the study group, most associations were not statistically significant (Table 6). However, although not significant for a 5% threshold, a tendency was observed for parent assistance while brushing, as children who were not assisted during dental brushing were mostly in the “Poor” and “Unsatisfactory” oral hygiene status. Children who were assisted by parents during brushing were more frequently found in the “Satisfactory” and “Good” oral hygiene status. A significant association was observed for dental floss use, as children who do not have this habit are mostly found in the “Poor” and “Unsatisfactory” oral hygiene status, while children who floss are mostly found in the “Good” oral hygiene status group.

For children in the control group, no statistically significant association was observed (Table 7).

**Figure 5:**
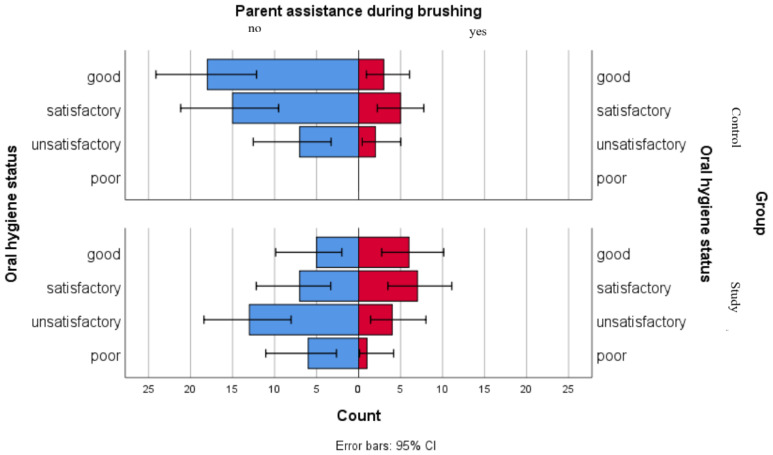
Comparative analysis of parent assistance during brushing and association with oral hygiene status categories.

We used a comparative graphical analysis to evaluate the distribution of parental assistance during brushing among oral hygiene status categories in both groups. The results point out that in the control group, distributions of oral hygiene statuses were similar regardless of parental assistance. In the study group, higher proportions of good and satisfactory oral hygiene statuses were observed among children receiving parental assistance, while children with unsatisfactory or poor oral hygiene statuses presented lower levels of parental assistance during brushing (Figure 5).

## 4. Discussion

The relationship between gingival and periodontal health and oral health-related quality of life (OHRQoL) was mostly studied in adults, the literature generally reporting that periodontal disease negatively affects life quality [25,26,27,28,29,30]. Some authors highlighted pain, psychological discomfort and functional limitations as main impacts of periodontal disease on the quality of life, without associations with disease severity [31], while others reported a greater alteration of OHRQoL linked to more severe periodontal conditions and that periodontal treatment can improve life quality [32,33,34,35].

In children and adolescents, the literature places gingival health amongst the main determinants of OHRQoL alterations, with negative impact on oral hygiene, esthetic function, daily activities and emotional well-being [36,37]. Some authors have reported a negative impact of gingivitis on the oral health-related quality of life in adolescents, independently from other oral conditions or socio-economic disparities [38]. Other studies described an infrequent correlation between periodontal disease and severe impact on daily activities of the children [39], while one recent study reports that mild gingival inflammation appears to have a limited effect on children’s perception over their quality of life [40]. Along with the direct effects on children, it appears that oral health issues associated with gingivitis also influence parents’ quality of life [41,42]. Overall, the available evidence indicates that gingivitis seems to have an important impact on the oral health-related quality of life in children and adolescents. Through its clinical manifestations such as bleeding, edema and erythema, gingival inflammation may compromise not only health parameters, but also the psychological factor, an essential component of OHRQoL [37,43,44].

Among the systemic conditions that can have significant effects on the oral cavity, leukemia frequently presents manifestations like gingival bleeding, gingival hypertrophy, ulcerations or opportunistic infections [45]. These changes can alter the oral health directly through pathological alterations of the oral mucosa and indirectly through reduced oral hygiene due to prioritization of general health concerns [46,47].

A recent study evaluating the oral health-related quality of life in children surviving oncological conditions reported a relatively good overall perception of these children of their own oral health state. Nevertheless, a higher prevalence of oral complications, such as ulcerations and halitosis were observed, especially in those diagnosed and treated in their early childhood, changes that can ultimately alter their quality of life. The authors emphasized the need for awareness programs, preventive approach and the necessity of education regarding the oncological treatment effects on the oral health state [48].

The results of our study indicate perceptual differences between the groups in reporting oral health-related complaints, as children with leukemia reported fewer concerns related to their smile appearance, the social impact of oral health issues as seen by peer difficulties, the presence of pain related to oral or dental problems, sleep troubles, concentration difficulties during school activities, school absenteeism caused by oro-dental pain and the presence of burning sensation. This pattern might reflect an adaptation of children suffering from leukemia to their systemic condition or a prioritization of general health concerns over oral health, rather than a lower prevalence or oral changes. The reported ulcerations, xerostomia and gingival bleeding showed a more complex distribution in children from the leukemia group, suggesting that although these children experience specific oral health issues, their self-reported psychosocial and functional impacts appear comparatively less pronounced. Systemic health prioritization and underestimation of oral changes might have influenced the responses, as well as parental proxy reporting, underlining the necessity to interpret the outcomes of perceived oral health in the general context of the chronic illness and its treatment. Similar observations are seen in the literature, as oral symptoms might be perceived by children and caregivers as less important in the circumstances of the systemic condition [48,49].

In our survey, the comparative analysis of items related to oral hygiene practices showed relatively few differences between children with leukemia and controls. Parental assistance during dental brushing varied between groups, with over one third of children with leukemia receiving assistance compared to approximately one fifth of children in the control group. This pattern aligns with previous reports underlining that parents of children with leukemia have a more active role in their daily oral hygiene routines [50,51]. A study in 2021 also underscored the necessity of prevention measures, along with dental health education and improvement of oral hygiene attitude, in children who exhibited a high prevalence of gingivitis [52].

When evaluating possible associations between gingival inflammation status and oral health as perceived by subjects in both groups, only xerostomia showed a statistically significant association in children with leukemia. This observation aligns with other studies that reported reduced salivary flow in relation to different degrees of gingival health in pediatric leukemia populations [53,54,55]. Apart from the limited statistically significant findings, the comparative graphical representations illustrate differences in the distribution of reported xerostomia, ulcerations and gingival bleeding between children with leukemia and those in the control group. The graphical patterns do not imply causal relationships, but may suggest variations in symptom reporting between groups, as children from the study group more frequently reported these symptoms across all gingival inflammation categories; this may reflect either the variability of symptom burdens, or differences in symptom perception and a better awareness or attentiveness to oral changes. Given the observational nature of our exploratory research, these findings should be interpreted as descriptive patterns rather than evidence of clinical causality. Overall, our findings align with previous reports, which show that despite the presence of various oral complaints and different degrees of gingival alterations, many children with leukemia report a relatively preserved oral health-related quality of life [56,57].

In our study, the exploratory association analysis between self-reported oral hygiene practices and oral hygiene status evaluated in children with leukemia suggests that not all reported behaviors closely correspond with clinical findings. Variables such as brushing frequency, type of toothbrush and mouthwash use were not associated with oral hygiene status, indicating that these responses might reflect habitual routines or different perceptions, rather than objective clinical conditions. Parental assistance during brushing showed distinctions across oral hygiene status categories; however, given the limited sample size, this observation should be cautiously interpreted. Dental flossing also showed an indicative pattern, with differing distributions among children in good, unsatisfactory and poor oral hygiene status categories. Overall, our findings suggest that self-reported oral hygiene practices provided by parents do not suggestively associate with clinically assessed oral hygiene status, and only certain habits such as dental flossing and parent assistance during brushing may be more closely reflected in clinical observations. For children in the control group, reported oral hygiene practices did not clearly distinguish between oral hygiene status categories. Descriptive graphical comparisons of parental assistance during brushing and oral hygiene status categories across both groups show varying distributions, particularly among children from the study group, without underlining causal relationships. These patterns might reflect difference in behavior reports or supplementary supervision practices which may be needed for children with leukemia in order to compensate for hygiene deficiencies driven by the disease itself or the administered treatment. Similar observations have been described in previous reports [46,57,58,59,60].

Our observations show that only 10 out of the 49 subjects in the leukemia group reported having received information about oral hygiene measures during the oncological treatment. Of these, four had consulted a dentist, while six had received guidance from the child’s attending physician during their hospitalization. This proportion indicates a relatively low rate of formal oral hygiene education and, considering that most advice was given by the general physician rather than dental professionals, this might reflect the limited integration of dental health care in the multidisciplinary medical team treating an oncologic patient. Our findings seem to align with other studies, which reported different barriers in oral care of leukemia subjects, including lack of dental awareness among the parents of children receiving chemotherapy [61] or inconsistent oral hygiene guidance, system-level support and caregiver disengagement [62].

The pediatric dentist should play an important role in the prevention and management of oral complications that may alter the quality of life of children with leukemia. Current literature focuses on the need for early diagnosis and prevention of oral changes occurring during the disease and following oncological treatment, as well as for caregiver education regarding oral hygiene measures during this critical and sensitive period. Furthermore, preventive dental assessment and oral health management prior to chemotherapy or radiotherapy regimens, along with the implementation of standardized oral health preventive protocols in all pediatric patients undergoing antineoplastic treatment, have been recommended in previous reports [55,63]. In this context, our findings might point out potential gaps in oral health education for families of children with leukemia, as well as a relatively narrow preventive involvement of dental professionals and oral health services for this vulnerable population. Establishing multidisciplinary protocols which can integrate pediatric dentists into the routine care of a child suffering from leukemia might optimize oral health outcomes and ensure a structured oral hygiene routine for these children, in line with existing recommendations for pediatric oncologic patients [64,65,66,67].

Recent studies identify oral mucositis as one of the most frequent oral complications in children undergoing oncological treatment, with a negative impact on their quality of life [49,68,69,70]. Mucosal ulcerations associated to mucositis often cause pain, feeding difficulties, as well as deglutition and phonation disturbances and may also influence survival rates [70]. In our study, although we have not directly evaluated mucositis in children undergoing leukemia therapy, we assessed reported modifications concerning xerostomia, burning sensation, ulcerations, gingival bleeding and evaluated gingival inflammation status using the Gingival Index. Although children with leukemia reported higher frequencies of these particular symptoms compared to subjects in the control group regardless of the gingival inflammation category, a key limitation of our study is the absence of a direct evaluation of mucositis as a distinct clinical entity.

Other limitations of our study should be acknowledged. The relatively small sample size may have reduced the statistical power and limit the strength of the observed results, along with the lack of adjustment for multiple comparisons and the absence of reported effect sizes. Also, sociodemographic confounders such as age, sex or environmental factors were not evaluated, as the assessment focused on certain symptomatology-driven aspects of oral health-related quality of life, with emphasis on gingival and periodontal alterations. Other not addressed potential confounders include the type of leukemia, treatment phase or time elapsed since diagnosis for the children in the study group, all of which may influence symptom perception and, subsequently, oral health-related quality of life. The parental proxy reporting might also be a limitation of our work, as responses could have been influenced and self-report bias might appear, particularly in the context of the evaluated systemic condition.

Additionally, the exploratory questionnaire we used was a personalized instrument derived from established tools (ECOHIS, COHIP, CPQ and POQL) and the lack of formal psychometric validation, including reliability and internal consistency testing, may affect the interpretability of the results. The psychosocial component of the questionnaire might not have comprehensively assessed the impact of priority shifts or adaptation processes of children with leukemia and their families in response to the severity of their systemic condition, as such factors might redirect the focus away from oral health and oral hygiene measures toward their general health concerns and disease-related weaknesses.

Although this study has several limitations, future research may address these issues and provide further insight into oral health-related quality of life in children with leukemia. The focus should follow the development and validation of standardized, disease-adapted OHRQoL instruments, specifically designed for this vulnerable pediatric population, considering variables such as leukemia type, treatment phase or sociodemographic factors, which would help clarify and interpret symptom perception, oral hygiene behaviors, as well as the clinical impact of gingivo-periodontal alterations. The use of such a validated, condition-specific tool on larger population samples might help develop individualized oral health management strategies and protocols specifically adapted to the needs of these children.

## 5. Conclusions

The results of our study suggest that self-reported oral health in children with leukemia may reflect the complex and intricate relationship between the systemic disease, treatment effects and individual perception of oral symptoms. Xerostomia seemed to be more closely aligned with the gingival inflammation status, while other oral symptoms such as ulcerations and gingival bleeding were frequently more reported regardless of gingival inflammation status category, suggesting that perception may not always correspond to clinical findings.

Observations related to parental assistance during dental brushing, as well as dental flossing habits in relation to the evaluated oral hygiene status, also suggest a potential influence of supportive oral hygiene practices on maintaining better oral health in children with leukemia.

Overall, our results underline the need for standardized protocols and individualized oral care management, as well as continuous monitoring, in order to support oral health and potentially enhance oral health-related quality of life in this vulnerable pediatric population.

## Figures and Tables

**Table 1:** Selection criteria for children in the study and control groups.

Subject	Inclusion Criteria	Exclusion Criteria
Study group	Age < 18 years old Any form of leukemia Any cancer treatment stage No periodontal treatment 6 months prior to the examination	Age > 18 years old Any periodontal treatment 6 months prior to the examination
Control group	Age < 18 years old No inflammatory systemic condition No periodontal treatment 6 months prior to the examination	

**Table 2:** Comparative summary table of the main variables considered in the first 10 items of the questionnaire (questions related to oral health as perceived by patients).

Variable	χ^2^ (df)	*p*-Value	Control Group *n* (%)	Study Group *n* (%)
Smile complaints	17.785 (3)	**0.000**	Never: 29 (58.0) Rarely: 8 (16.0) Quite often: 7 (14.0) Often: 6 (12.0)	Never: 45 (91.8) Rarely: 4 (8.2) Quite often: 0 (0.0) Often: 0 (0.0)
Peer difficulties	5.835 (1)	**0.016**	Never: 42 (84.0) Rarely: 8 (16.0)	Never: 48 (98.0) Rarely: 1 (2.0)
Pain	10.775 (3)	**0.013**	Never: 15 (30.0) Rarely: 23 (46.0) Quite often: 6 (12.0) Often: 6 (12.0)	Never: 25 (51.0) Rarely: 15 (30.6) Quite often: 9 (18.4) Often: 0 (0.0)
Sleep disturbances	26.478 (3)	**0.000**	Never: 24 (48.0) Rarely: 11 (22.0) Quite often: 8 (16.0) Often: 7 (14.0)	Never: 46 (93.9) Rarely: 3 (6.1) Quite often: 0 (0.0) Often: 0 (0.0)
Concentration difficulties	27.720 (3)	**0.000**	Never: 28 (56.0) Rarely: 7 (14.0) Quite often: 7 (14.0) Often: 8 (16.0)	Never: 49 (100.0) Rarely: 0 (0.0) Quite often: 0 (0.0) Often: 0 (0.0)
School absenteeism	27.720 (3)	**0.000**	Never: 28 (56.0) Rarely: 11 (22.0) Quite often: 5 (10.0) Often: 6 (12.0)	Never: 49 (100.0) Rarely: 0 (0.0) Quite often: 0 (0.0) Often: 0 (0.0)
Burning sensation	7.004 (3)	0.072 *	Never: 25 (50.0) Rarely: 14 (28.0) Quite often: 5 (10.0) Often: 6 (12.0)	Never: 32 (65.3) Rarely: 12 (24.5) Quite often: 5 (10.2) Often: 0 (0.0)
Gingival bleeding	3.766 (2)	0.152	Never: 20 (40.0) Rarely: 18 (36.0) Quite often: 12 (24.0)	Never: 29 (59.2) Rarely: 13 (26.5) Quite often: 7 (14.3)
Ulcerations	18.678 (4)	**0.001**	Never: 22 (44.0) Rarely: 22 (44.0) Quite often: 0 (0.0) Often: 6 (12.0) Very often: 0 (0.0)	Never: 10 (20.4) Rarely: 20 (40.8) Quite often: 11 (22.5) Often: 5 (10.2) Very often: 3 (6.1)
Dry mouth	5.395 (2)	0.067 *	Never: 17 (34.0) Rarely: 21 (42.0) Quite often: 12 (24.0)	Never: 28 (57.1) Rarely: 14 (28.6) Quite often: 7 (14.3)

χ^2^ = Pearson’s Chi-square statistic; df = degrees of freedom; * Indicates significance at the 10% level (*p* < 0.10).

**Table 3:** Comparative summary table of main variables considered in the second section of the questionnaire (5 questions related to oral hygiene as perceived by patients).

Variable	χ^2^ (df)	*p*-Value	Category	Control Group *n* (%)	Study Group *n* (%)
Brushing frequency	0.046 (2)	0.831	Never brushes	6 (12.0)	5 (11.3)
			1/day	23 (46.0)	24 (48.9)
			2/day	21 (42.0)	20 (40.8)
			Total valid	44 (100)	44 (100)
Parent assistance during brushing	3.417 (1)	0.065 *	No	40 (80.0)	31 (63.3)
			Yes	10 (20.0)	18 (36.7)
			Total	50 (100)	49 (100)
Brush type	4.119 (3)	0.249	None	8 (16.0)	5 (10.2)
			Manual	27 (54.0)	33 (67.3)
			Electric	8 (16.0)	9 (18.4)
			Both	7 (14.0)	2 (4.1)
			Total	50 (100)	49 (100)
Mouthwash use	3.198 (2)	0.202	No	24 (48.0)	32 (65.3)
			Yes	14 (28.0)	8 (16.3)
			Occasionally	12 (24.0)	9 (18.4)
			Total	50 (100)	49 (100)
Dental floss use	3.312 (1)	0.069 *	No	38 (76.0)	44 (89.8)
			Yes	12 (24.0)	5 (10.2)
			Total	50 (100)	49 (100)

χ^2^ = Pearson’s Chi-square statistic; df = degrees of freedom; * Indicates significance at the 10% level (*p* < 0.10).

**Table 4:** Association between gingival inflammation state (mild/moderate/severe) and oral health as perceived by subjects in the study group.

Question	Category	Mild	Moderate	Severe	Total *n* (%)	χ^2^	*p*-Value
Smile complaints	Never	20	13	12	45 (91.8)	0.049	0.976
	Rarely	2	1	1	4 (8.2)		
Peer difficulties	Never	22	14	12	48 (98.0)	2.827	0.243
	Rarely	0	0	1	1 (2.0)		
Pain	Never	13	6	6	25 (51.0)	1.220	0.875
	Rarely	6	5	4	15 (30.6)		
	Quite often	3	3	3	9 (18.4)		
Sleep disturbances	Never	21	14	11	46 (93.9)	2.949	0.229
	Rarely	1	0	2	3 (6.1)		
Concentration difficulties	Never	22	14	13	49 (100)	–	–
School absenteeism	Never	22	14	13	49 (100)	–	–
Burning sensation	Never	17	8	7	32 (65.3)	3.412	0.491
	Rarely	3	4	5	12 (24.5)		
	Quite often	2	2	1	5 (10.2)		
Gingival bleeding	Never	16	7	6	29 (59.2)	4.149	0.386
	Rarely	5	4	4	13 (26.5)		
	Quite often	1	3	3	7 (14.3)		
Ulcerations	Never	7	2	1	10 (20.4)	10.969	0.203
	Rarely	10	5	5	20 (40.8)		
	Quite often	5	2	4	11 (22.4)		
	Often	0	3	2	5 (10.2)		
	Very often	0	2	1	3 (6.1)		
Dry mouth	Never	14	11	3	28 (57.1)	10.852	**0.028**
	Rarely	4	3	7	14 (28.6)		
	Quite often	4	0	3	7 (14.3)		

χ^2^ = Pearson’s Chi-square statistic.

**Table 5:** Association between gingival inflammation state (mild/moderate/severe) and oral health as perceived by subjects in the control group.

Question	Category	Mild	Moderate	Total *n* (%)	χ^2^	*p*-Value
Smile complaints	Never	23	6	29 (58)	2.798	0.424
	Rarely	7	1	8 (16)		
	Quite often	7	0	7 (14)		
	Often	4	2	6 (12)		
Peer difficulties	Never	36	6	42 (84)	2.454	0.117
	Rarely	5	3	8 (16)		
Pain	Never	13	2	15 (30)	2.712	0.438
	Rarely	18	5	23 (46)		
	Quite often	6	0	6 (12)		
	Often	4	2	6 (12)		
Sleep disturbances	Never	21	3	24 (48)	3.674	0.299
	Rarely	10	1	11 (22)		
	Quite often	5	3	8 (16)		
	Often	5	2	7 (14)		
Concentration difficulties	Never	24	4	28 (56)	1.123	0.772
	Rarely	5	2	7 (14)		
	Quite often	6	1	7 (14)		
	Often	6	2	8 (16)		
School absenteeism	Never	23	5	28 (56)	1.746	0.627
	Rarely	8	3	11 (22)		
	Quite often	5	0	5 (10)		
	Often	5	1	6 (12)		
Burning sensation	Never	21	4	25 (50)	0.200	0.978
	Rarely	11	3	14 (28)		
	Quite often	4	1	5 (10)		
	Often	5	1	6 (12)		
Gingival bleeding	Never	17	3	20 (40)	0.354	0.838
	Rarely	14	4	18 (36)		
	Quite often	10	2	12 (24)		
Ulcerations	Never	19	3	22 (44)	0.624	0.732
	Rarely	17	5	22 (44)		
	Often	5	1	6 (12)		
Dry mouth	Never	15	2	17 (34)	2.556	0.279
	Rarely	18	3	21 (42)		
	Quite often	8	4	12 (24)		

χ^2^ = Pearson’s Chi-square statistic.

**Table 6:** Association between oral hygiene status (poor/unsatisfactory/satisfactory/good) and oral hygiene as perceived by subjects in the study group.

Question	Category	Poor	Unsatisfactory	Satisfactory	Good	Total *n* (%)	Pearson Chi-Square	*p*-Value
Brushing frequency	Never brushes	1	2	1	1	5 (10.2)	0.766	0.993
	1	4	8	7	5	24 (48.9)		
	2	2	7	6	5	20 (40.8)		
Parent assistance during brushing	No	6	13	7	5	31 (63.2)	5.355	0.148 *
	Yes	1	4	7	6	18 (36.7)		
Brush type	None	1	2	1	1	5 (10.2)	6.397	0.700
	Manual	3	10	12	8	33 (67.3)		
	Electric	2	4	1	2	9 (18.3)		
	Both	1	1	0	0	2 (4.1)		
Mouthwash use	No	5	10	11	6	32 (65.3)	2.562	0.861
	Yes	1	3	2	2	8 (16.3)		
	Occasionally	1	4	1	3	9 (18.3)		
Dental floss use	No	6	17	14	7	44 (89.7)	11.865	**0.008**
	Yes	1	0	0	4	5 (10.2)		

* Indicates significance at the 10% level (*p* < 0.10).

**Table 7:** Association between oral hygiene status (unsatisfactory/satisfactory/good) and oral hygiene as perceived by subjects in the control group.

Question	Category	Unsatisfactory	Satisfactory	Good	Total *n* (%)	χ^2^	*p*-Value
Brushing frequency	Never brushes	1	5	0	6 (12)		
	1/day	6	8	9	23 (46)	8.598	0.072
	2/day	2	7	12	21 (42)		
Parent assistance during brushing	No	7	15	18	40 (80)	0.769	0.681
	Yes	2	5	3	10 (20)		
Brush type	None	1	6	1	8 (16)	9.095	0.168
	Manual	6	7	14	27 (54)		
	Electric	2	4	2	8 (16)		
	Both	0	3	4	7 (14)		
Mouthwash use	No	3	11	10	24 (48)	7.279	0.122
	Yes	2	3	9	14 (28)		
	Occasionally	4	6	2	12 (24)		
Dental floss use	No	7	14	17	38 (72)	0.693	0.707
	Yes	2	6	4	12 (24)		

χ^2^ = Pearson’s Chi-square statistic.

## Data Availability

The original contributions presented in this study are included in the article and Supplementary Materials. Further inquiries can be directed to the corresponding author.

## Supplementary Materials

The following supporting information can be downloaded at: https://www.mdpi.com/article/10.3390/jpm16020084/s1, Figure S1: Parents questionnaire for children in the control group; Figure S2: Parents questionnaire for children in the study group.

## Abbreviations

The following abbreviations are used in this manuscript:

OHRQoL	Oral health-related quality of life
ECOHIS	Early Childhood Oral Health Impact Scale
COHIP	Child Oral Health Impact Profile
CPQ	Child Perceptions Questionnaire
POQL	Pediatric Oral Health-related Quality of Life Questionnaire
Child-OIDP	Child—Oral Impact on Daily Performance
OHI-S	Oral Hygiene Index—Simplified
GI	Gingival Index
