# Supplementary material for: Assessment of Oral Health-Related Quality of Life in Children with Leukemia and Gingival Inflammation
Source: J Pers Med. 2026 Feb 2;16(2):84. doi: 10.3390/jpm16020084 (PMC12941404; doi:10.3390/jpm16020084)
Supplement: Supplementary file 1 [file jpm-16-00084-s001.zip › jpm-4056590-supplementary.pdf]

Supplementary Materials

PARENTS QUESTIONNAIRE

You are kindly asked to read this questionnaire carefully before answering and to ask any questions if something is unclear.

The questionnaire consists of two parts, each containing several questions. You are asked to check the box corresponding to the appropriate answer.

**Questions regarding oral health**	**Answer**				
	**Very often**	**Often**	**Quite often**	**Rarely**	**Never**
Does your child complain of his smile?					
Does your child have problems in his/her relationship with friends/peers because of his smile (is he/she bullied/ashamed or shy/does not smile because of his/her teeth)?					
Does your child complain of pain in the oral cavity (lips, tongue, gums, teeth)?					
Does your child wake up from sleep because of this pain?					
Does your child complain of difficulties concentrating in school because of this pain?					
Does your child miss school because of this pain?					
Does your child complain of a burning sensation in the oral cavity (lips, tongue, gums)?					
Does your child complain of bleeding gums?					
Does your child complain of ulcerations (such as canker sores) in the oral cavity (lips, tongue, gums)?					
Does your child complain of dry mouth sensation?					
**Questions regarding oral hygiene**	**Answer**				
How many times a day does your child brush his/her teeth?					
Do you help / assist your child brushing teeth?					
Does your child use a manual or electric tooth brush?					
Does your child use mouthwash? If so, how many times a day?					
Does your child use dental floss? If so, how many times a day?					

Figure S1. Parents questionnaire for children in the control group.

PARENTS QUESTIONNAIRE

You are kindly asked to read this questionnaire carefully before answering and to ask any questions if something is unclear.

The questionnaire consists of two parts, each containing several questions. You are asked to check the box corresponding to the appropriate answer.

**Questions regarding oral health**	**Answer**				
	**Very often**	**Often**	**Quite often**	**Rarely**	**Never**
Does your child complain of his smile?					
Does your child have problems in his/her relationship with friends/peers because of his smile (is he/she bullied/ashamed or shy/does not smile because of his/her teeth)?					
Does your child complain of pain in the oral cavity (lips, tongue, gums, teeth)?					
Does your child wake up from sleep because of this pain?					
Does your child complain of difficulties concentrating in school because of this pain?					
Does your child miss school because of this pain?					
Does your child complain of a burning sensation in the oral cavity (lips, tongue, gums)?					
Does your child complain of bleeding gums?					
Does your child complain of ulcerations (such as canker sores) in the oral cavity (lips, tongue, gums)?					
Does your child complain of dry mouth sensation?					
**Questions regarding oral hygiene**	**Answer**				
How many times a day does your child brush his/her teeth?					
Do you help / assist your child brushing teeth?					
Does your child use a manual or electric tooth brush?					
Does your child use mouthwash? If so, how many times a day?					
Does your child use dental floss? If so, how many times a day?					
"Have you received any information about how your child's oral hygiene should be maintained during anti-leukemic treatment? If yes, from whom?"					

Figure S2. Parents questionnaire for children in the study group.
